# Network architecture determines delay robustness in the spindle assembly checkpoint

**DOI:** 10.1038/s41598-026-61538-y

**Published:** 2026-07-14

**Authors:** Bashar Ibrahim

**Affiliations:** 1 Department of Mathematics and Computer Science, Friedrich Schiller University Jena, Fürstengraben, 07743 Jena, Germany; 2 European Virus Bioinformatics Center, Leutragraben 1, 07743 Jena, Germany

**Keywords:** Delay differential equations, Nonlinear dynamics, Bifurcation theory, Spindle assembly checkpoint, Biophysics, Computational biology and bioinformatics, Mathematics and computing, Physics, Systems biology

## Abstract

The spindle assembly checkpoint (SAC) ensures accurate chromosome segregation during mitosis by preventing premature activation of the anaphase-promoting complex/cyclosome (APC/C). Despite its critical role in maintaining genomic stability, most mathematical models of the SAC treat underlying biochemical processes as instantaneous, neglecting experimentally observed delays arising from molecular activation, complex assembly, and intracellular transport. How such temporal structure interacts with network architecture to shape checkpoint dynamics remains unclear. Here, we develop a distributed-delay framework and incorporate experimentally motivated delays into multiple mechanistic SAC architectures. Using a gamma-chain formulation, we perform systematic stability and bifurcation analyses across representative models. We find that biologically realistic delays fundamentally reorganize system dynamics, partitioning SAC architectures into two distinct classes: delay-robust designs that preserve strong APC/C inhibition, and delay-sensitive designs in which checkpoint control collapses. Motivated by this classification, we introduce a bistable template architecture that combines mechanistic Mad2 templating with an autocatalytic feedback loop. This design maintains bistability and high inhibition across a broad range of physiological delays and remains resilient under stochastic perturbations. These results identify network architecture as a key determinant of robustness to molecular timing and demonstrate that distributed delays can stabilize, rather than destabilize, checkpoint function by enabling temporal integration and memory-like behavior. More broadly, this work establishes delay-aware design principles for biochemical decision-making systems in which intermediate processes are intrinsically time-distributed.

## Introduction

Faithful chromosome segregation during mitosis is essential for maintaining genomic integrity. Perturbations in this process lead to chromosome missegregation and aneuploidy, a defining feature of many cancers and developmental disorders^1,2^. Because several anticancer therapies exploit vulnerabilities in mitotic timing, understanding how mitotic progression is regulated remains a central objective in both cell biology and medicine. A key element of this control is the spindle assembly checkpoint (SAC), which delays anaphase onset until all kinetochores establish stable, bipolar attachments to spindle microtubules^3,4^. Checkpoint activation suppresses the anaphase-promoting complex/cyclosome (APC/C) by preventing its activation by Cdc20 through formation of the mitotic checkpoint complex (MCC), a stoichiometric assembly of Cdc20, Mad2, BubR1, and Bub3^5,6^.

SAC signaling is initiated at unattached kinetochores, where the Mad1:C-Mad2 complex catalyzes the conversion of open Mad2 (O-Mad2) into its closed, active conformation (C-Mad2) via a templated mechanism^7,8^. Activated C-Mad2 subsequently binds Cdc20, promoting MCC assembly and amplifying a localized attachment error into a cell-wide APC/C-inhibitory signal^9,10^. For effective checkpoint enforcement, this inhibitory signal must propagate through the cytoplasm, overcoming spatial constraints, molecular crowding, and transport along spindle-associated structures^11,12^. These biochemical and physical processes necessarily introduce finite reaction and transport times at multiple regulatory stages.

Similar Mad2-dependent checkpoint mechanisms have also been extensively characterized in yeast systems, where conserved templating and feedback interactions contribute to robust mitotic surveillance^3^.

Most mathematical descriptions of the SAC idealize these processes as instantaneous. Early ordinary differential equation (ODE) models demonstrated that Cdc20 sequestration and feedback amplification can generate switch-like, bistable inhibition of APC/C activity^13,14^. Subsequent frameworks incorporated spatial compartmentalization^15^, stochastic fluctuations^16,17^, or detailed templated activation mechanisms^18^, providing increasingly refined mechanistic insight. Nevertheless, these models typically neglect experimentally observed delays associated with Mad2 conformational activation, MCC maturation, and cytoplasmic signal propagation, despite the fact that human mitosis lasts only $\sim 20$ minutes and delays on the order of tens of seconds to minutes are therefore non-negligible.

Experimental measurements indeed indicate pronounced non-instantaneous behavior in SAC signaling. Mad2 activation and MCC formation require approximately 40–80 seconds^10,19^, while cytoplasmic transport and spindle geometry introduce additional delays on the order of minutes^5,12^. Single-cell imaging studies further reveal substantial cell-to-cell variability in mitotic duration^20^, suggesting that timing variability arises not only from molecular noise but also from delayed regulatory feedback. These observations highlight that temporal structure is an intrinsic component of SAC regulation rather than a secondary effect.

From a theoretical perspective, delayed feedback is well known to profoundly alter the qualitative behavior of biochemical networks. Classical studies have shown that time delays can induce multistability, sharpen switch-like responses, generate hysteresis, or destabilize steady states^21–28^. Recent studies have further demonstrated that delayed feedback can reorganize nonlinear biochemical dynamics and generate complex bifurcation structures in regulatory systems^29^. However, despite these insights, delay-induced phenomena have not been systematically investigated in SAC modeling, particularly in relation to how network architecture modulates sensitivity to molecular timing^30^.

In particular, it remains unclear whether all SAC architectures respond similarly to biologically realistic delays, or whether robustness to temporal dispersion is an emergent property of specific network topologies. Addressing this question is essential for understanding how cells maintain reliable checkpoint control despite intrinsically delayed biochemical processes.

To address this gap, we develop a mechanistically grounded delay–differential equation (DDE) framework incorporating three experimentally motivated delays: (i) Mad2 activation ($\tau _{\text {Mad2}} \approx 75$ s), (ii) MCC maturation ($\tau _{\text {MCC}} \approx 45$ s), and (iii) cytoplasmic signal propagation ($\tau _{\text {transport}} \approx 105$ s)^5,10,12,31^. These delays are embedded into four canonical SAC architectures–*Doncic Emitted*, *Lohel Implicit*, *Lohel Explicit*, and the mechanistic *Mad2 Template*—and extended to a new *Bistable Template* model that combines template-driven activation with an autocatalytic APC/C inhibition loop.

Our analysis provides a systematic classification of SAC architectures based on their robustness to molecular timing. Distributed delays reorganize the phase space and separate SAC architectures into two distinct classes: delay-robust designs that preserve strong APC/C inhibition, and delay-sensitive designs in which checkpoint control collapses. Furthermore, delays induce saddle-node bifurcations, expand bistable regimes, and increase hysteresis width. The Bistable Template model, in particular, maintains bistability and robust checkpoint activity across wide delay ranges and remains functional under stochastic perturbations, indicating that delayed feedback can, under appropriate architectural conditions, reinforce rather than compromise robustness.

Together, these results identify network architecture as a key determinant of robustness to molecular timing and establish temporal organization as a core regulatory principle of SAC control. More broadly, this work provides a general framework for analyzing delay-regulated signaling in mitotic checkpoints and other cellular decision-making systems governed by intrinsically time-distributed processes.

## Mathematical framework

### Distributed delay differential equations

To model the temporal structure of spindle assembly checkpoint (SAC) signaling, we formulate a delay–differential equation (DDE) framework in which key biochemical species evolve under delayed regulatory interactions. In this setting, $x(t)\in \mathbb {R}^m$ denotes the vector of molecular concentrations (e.g., Cdc20, MCC, APC/C complexes), and the system takes the general form

1 $$\begin{aligned} \frac{dx}{dt} = f\big (x(t),\,x(t-\tau )\big ), \end{aligned}$$

where $\tau>0$ represents a characteristic delay associated with biochemical activation, complex assembly, or intracellular transport.

In biological systems, however, reaction and transport times are rarely discrete. To capture their intrinsic variability, we replace discrete delays with *distributed* delays, yielding

2 $$\begin{aligned} \frac{dx}{dt} = f\left( x(t),\,\int _{0}^{\infty } g(s)\,x(t-s)\,ds\right) , \end{aligned}$$

where the kernel

3 $$\begin{aligned} g(s)=\frac{(n/\tau )^n}{\Gamma (n)}\, s^{n-1} e^{-ns/\tau } \end{aligned}$$

is a gamma density with shape parameter *n* and rate $n/\tau$^32,33^. This formulation incorporates temporal dispersion directly into the dynamics, allowing delayed biochemical processes to act as smooth memory terms while preserving analytical tractability.

### Gamma-chain approximation

To enable efficient numerical simulation and bifurcation analysis, we approximate the distributed delay using a *gamma-chain representation*. The convolution term in (2) is replaced by a sequence of *n* auxiliary variables:

4 $$\begin{aligned} \frac{dx_1}{dt}&= \frac{n}{\tau }\big (x(t)-x_1(t)\big ), \end{aligned}$$

5 $$\begin{aligned} \frac{dx_i}{dt}&= \frac{n}{\tau }\big (x_{i-1}(t)-x_i(t)\big ), \qquad i=2,\ldots ,n, \end{aligned}$$

6 $$\begin{aligned} x_{\tau }(t)&\approx x_n(t), \end{aligned}$$

which yields a finite-dimensional ODE system equivalent to a gamma-distributed delay.

As *n* increases, this approximation converges to the exact distributed-delay formulation with error of order $O(n^{-1/2})$^32,33^. In all simulations, we use $n=5$ (coefficient of variation $\approx 0.45$), consistent with experimentally observed variability in SAC timing processes. To evaluate the sensitivity of the distributed-delay approximation to the gamma-chain order, we additionally examined larger values including $n=10$ and $n=50$. These analyses confirmed that the qualitative dynamical behavior and the classification into delay-robust and delay-sensitive architectures remained unchanged as *n* increased (Supplementary Figure S1 and Supplementary Table S1). As expected, larger *n* values produced narrower delay distributions approaching the discrete-delay limit without altering the principal conclusions of the study.

**Biological Interpretation**

The gamma-chain formulation establishes a direct correspondence between model parameters and molecular processes:

1. Each chain represents a sequence of intermediate biochemical steps underlying activation or transport.
2. Mad2 activation, MCC maturation, and cytoplasmic propagation are modeled as temporally distributed processes rather than instantaneous events.
3. The resulting dynamics retain key qualitative features of SAC regulation, including bistability, threshold behavior, and memory effects (Table 1).

**Table 1:** Biological interpretation of distributed delays used in SAC models.

Delay	Biological process	Affected module
$\tau _{\textrm{Mad2}}$	Mad2 conformational activation	Mad2 templating
$\tau _{\textrm{MCC}}$	MCC maturation and assembly	MCC formation
$\tau _{\textrm{transport}}$	Cytoplasmic signal propagation	APC/C inhibitory signaling

**Computational Advantages.**

The gamma-chain approximation transforms the infinite-dimensional delay system into a finite set of ODEs, enabling the use of standard continuation and bifurcation tools such as MATCONT and AUTO. This avoids numerical instability associated with direct DDE solvers while preserving the essential dynamical structure.

### Stability and accuracy

The gamma-chain approximation preserves the stability properties of the underlying distributed-delay system. In particular, the characteristic equation

7 $$\begin{aligned} \det \!\left( \lambda I - A - B\left( \frac{n/\tau }{\lambda +n/\tau }\right) ^n\right) =0 \end{aligned}$$

converges to the discrete-delay form

8 $$\begin{aligned} \det \!\left( \lambda I - A - B e^{-\lambda \tau }\right) =0 \end{aligned}$$

as $n\rightarrow \infty$^32,34^. Thus, equilibria, local stability, and bifurcation points are accurately captured, providing a rigorous basis for analyzing delay-induced transitions.

### Application to the spindle assembly checkpoint

We apply this framework to mechanistic SAC models by incorporating three experimentally grounded delays:

9 $$\begin{aligned} \tau _{\text {Mad2}}&= 75~\text {s}, \end{aligned}$$

10 $$\begin{aligned} \tau _{\text {MCC}}&= 45~\text {s}, \end{aligned}$$

11 $$\begin{aligned} \tau _{\text {transport}}&= 105~\text {s}. \end{aligned}$$

Each delay is implemented using a five-stage gamma chain, introducing 15 auxiliary variables into each SAC model. These delayed processes act on specific reaction modules, including Mad2 conformational conversion, MCC assembly, and cytoplasmic signal propagation.

The framework is applied consistently across multiple SAC architectures— *Doncic Emitted*, *Lohel Implicit*, *Lohel Explicit*, and *Mad2 Template*—as well as the proposed *Bistable Template* model. This unified formulation enables direct comparison of how network architecture modulates sensitivity to temporal delays.

Across physiologically realistic parameter ranges, distributed delays induce saddle-node bifurcations, expand bistable regimes, and increase hysteresis width. These effects provide the mechanistic basis for the classification of SAC architectures into delay-robust and delay-sensitive regimes presented in the Results section.

### Representative model formulation

To make the above framework explicit, we present the governing equations for the proposed *Bistable Template* model, which combines mechanistic Mad2 templating with autocatalytic APC/C inhibition. The model tracks the concentrations of key molecular species: closed Mad2 ($M^*$), free Cdc20 (*C*), mitotic checkpoint complex (*M*), active APC/C (*A*), and inhibited APC/C ($A_M$).

The dynamics are governed by the following system of ordinary differential equations:

12 $$\begin{aligned} \frac{dM^*}{dt}&= k_{\textrm{temp}}\,\textrm{KinU}\,(M_{\textrm{tot}} - M^*) - k_{\textrm{form}}\,M^*C, \end{aligned}$$

13 $$\begin{aligned} \frac{dM}{dt}&= k_{\textrm{form}}\,M^*C - k_{\textrm{bind}}\,M A + k_{\textrm{unbind}}\,A_M, \end{aligned}$$

14 $$\begin{aligned} \frac{dA_M}{dt}&= k_{\textrm{bind}}\,M A - k_{\textrm{unbind}}\,A_M - k_{\textrm{fb}}\,A_M, \end{aligned}$$

15 $$\begin{aligned} \frac{dA}{dt}&= -k_{\textrm{bind}}\,M A + k_{\textrm{unbind}}\,A_M + k_{\textrm{fb}}\,A_M, \end{aligned}$$

16 $$\begin{aligned} \frac{dC}{dt}&= -k_{\textrm{form}}\,M^*C + k_{\textrm{fb}}\,A_M, \end{aligned}$$

subject to conservation constraints

17 $$\begin{aligned} A_{\textrm{tot}}&= A + A_M, \end{aligned}$$

18 $$\begin{aligned} C_{\textrm{tot}}&= C + M + A_M. \end{aligned}$$

Here, $\textrm{KinU}$ denotes the number of unattached kinetochores, which drives Mad2 activation, $M_{\textrm{tot}}$ is the total Mad2 concentration, and all rate constants correspond to biochemical processes defined in the Materials and Methods section.

Delayed processes enter the system through gamma-chain variables associated with Mad2 activation, MCC maturation, and cytoplasmic transport. Specifically, terms involving $M^*$ and *M* are replaced by their delayed counterparts using the distributed-delay formulation described above.

**Fig. 1:**
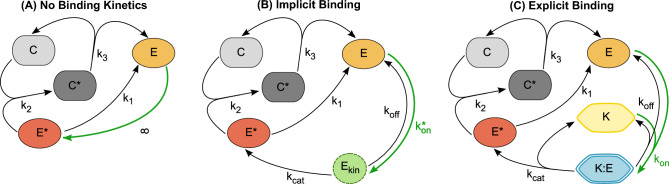
Canonical SAC model variants arranged by increasing mechanistic complexity. **(A)** Doncic Emitted: idealized, instantaneous kinetochore activation. **(B)** Lohel Implicit: finite on/off rates without explicit saturation. **(C)** Lohel Explicit: Michaelis–Menten binding with saturable intermediates. Green arrows indicate kinetochore-localized reactions; blue boxes denote spatially restricted species. The level of mechanistic detail increases from (A) to (C).

This system captures the core feedback structure underlying bistable checkpoint control: templated Mad2 activation generates MCC, which inhibits APC/C, while an autocatalytic feedback term ($k_{\textrm{fb}}$) reinforces Cdc20 sequestration. The resulting nonlinear interactions give rise to bistability, hysteresis, and delay-dependent bifurcations (Fig. 1).

All other SAC architectures analyzed in this work follow analogous formulations with architecture-specific reaction terms. The complete set of equations for all models is provided in Additional information.

## Results

### Distributed delays stratify SAC architectures into timing-sensitivity classes

We first examined how physiologically realistic biochemical delays influence spindle assembly checkpoint (SAC) performance across established model architectures, with a particular focus on whether robustness to molecular timing is determined by network structure. To this end, we incorporated three experimentally constrained delays—Mad2 activation, MCC maturation, and cytoplasmic transport—into five representative SAC models. These comprise three binding-kinetics formulations (Doncic Emitted, Lohel Implicit, Lohel Explicit) and two mechanistic, template-based designs (Mad2 Template and the newly introduced Bistable Template). By embedding identical delay distributions into each system, we isolate the role of interaction topology in determining whether checkpoint control is preserved, bistable switching occurs, or inhibition collapses.

**Fig. 2:**
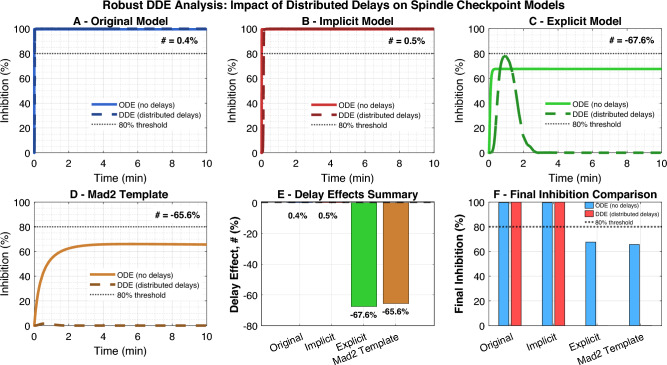
Distributed delays discriminate robust and fragile SAC architectures.**(A–D)** Time courses comparing ODE (solid) and distributed-delay (dashed) dynamics for the four canonical variants following kinetochore activation at $t=0$. **(E)** Delay-induced change in steady-state inhibition ($\Delta$ inhibition). **(F)** Final inhibition across architectures. Delay-robust models retain $>\!80\%$ inhibition, whereas delay-sensitive designs lose checkpoint function under the same physiological delays.

The canonical architectures—**Doncic Emitted**, **Lohel Implicit**, and **Lohel Explicit**—exhibited markedly different behaviors once realistic delays were introduced. Time-course simulations showed that the Doncic Emitted and Lohel Implicit models retained strong checkpoint activity, with APC/C inhibition remaining above 95% (quantified via the APC/C:MCC fraction; see Materials and Methods) across all tested delay conditions. In contrast, the Lohel Explicit and Mad2 Template architectures failed to sustain MCC accumulation and relaxed toward near-complete loss of APC/C inhibition under identical delay settings (Fig. 2). A quantitative comparison of steady-state inhibition between ODE and distributed-delay formulations confirmed this dichotomy: in delay-robust designs, final inhibition was essentially unchanged, whereas delay-sensitive architectures experienced a pronounced decline (Table 2).

These observations partition the SAC architectures, within the explored parameter regime, into two distinct *timing-sensitivity classes*. In delay-robust systems, steady states corresponding to strong APC/C inhibition persist under physiologically realistic delays and possess large basins of attraction. In contrast, delay-sensitive designs undergo qualitative changes in their phase portrait, losing the inhibited steady state and collapsing to a low-inhibition regime. Because all models are subjected to identical delay parameters, these differences arise from interaction topology rather than kinetic fine-tuning, demonstrating that robustness to molecular timing is an emergent dynamical property encoded in network structure. These results demonstrate that robustness to molecular timing is not universal, but is instead determined by network architecture, revealing a structural origin of delay tolerance in SAC signaling.

**Notation for Figure **1. We denote free APC/C by *E*, APC/C bound to Cdc20 by *C*, inhibited APC/C by $C^*$, and a coarse-grained inhibited pool by $E^*$. In the explicit-binding formulation, *K* represents the APC/C:Cdc20 intermediate and $K\!:\!E$ its inhibited complex. These schematics highlight binding architecture only; complete reaction networks are provided in the Materials and Methods.

Throughout this work, “checkpoint inhibition” refers to the fraction of APC/C residing in MCC-bound or otherwise inhibited states (APC/C:MCC or its coarse-grained analog), which serves as our primary measure of SAC activation strength.

**Table 2:** Quantitative classification of SAC architectures by timing sensitivity.

Model	Delay Effect	Final Inhibition	Robustness	Class
	($\Delta$%)	(ODE/DDE)	Score	
Doncic Emitted	+0.4%	98%/98%	0.996	Delay-robust
Lohel Implicit	+0.5%	97%/97%	0.995	Delay-robust
Lohel Explicit	$-67.6\%$	67%/0%	0.000	Delay-sensitive
Mad2 Template	$-65.6\%$	65%/0%	0.000	Delay-sensitive

### A delay-aware bistable template architecture stabilizes SAC control

To test whether delay sensitivity can be overcome through architectural design, we constructed a **Bistable Template** model that exploits, rather than is destabilized by, temporal delays. We developed this model by augmenting the mechanistic Mad2 Template framework with an explicit autocatalytic feedback loop. The upstream kinetochore module—KinU/KinA regulation and Mad1:C-Mad2–mediated conversion of O-Mad2 to C-Mad2—remains unchanged, ensuring that differences in delay robustness arise solely from APC/C regulation rather than altered kinetochore signaling. The Bistable Template constitutes a mechanistic extension of template-based SAC designs rather than a modification of the binding-kinetics variants in Fig. 1. Its defining features are: (i) templated generation of C-Mad2, (ii) explicit MCC formation, and (iii) an autocatalytic reinforcement loop in which inhibited APC/C promotes further Cdc20 sequestration. This wiring introduces a strong positive-feedback motif capable of generating bistable switching under biologically realistic delays.

In this formulation, the inhibitory step

$$\mathrm {MCC + APC/C \rightleftharpoons APC/C{:}MCC}$$

represents a coarse-grained description of the experimentally established targeting of APC/C:Cdc20. Earlier SAC models that explicitly tracked APC/C:Cdc20 exhibit similar hysteresis and switch-like behavior^16,35^, validating this reduced representation.

Embedding the three distributed delays into the Bistable Template yields dynamics that are markedly more robust than those of delay-sensitive architectures. The model sustains high MCC levels and maintains $>\!90\%$ APC/C inhibition across the entire sampled delay range, whereas the Lohel Explicit and Mad2 Template models collapse under identical conditions. One-parameter continuation of total MCC reveals a saddle-node bifurcation separating low- and high-inhibition states, with the delayed system exhibiting an expanded bistable interval relative to the ODE formulation. Monte Carlo sampling with 15% parameter variability confirms that bistability and high inhibition are structurally stable features of this architecture rather than artifacts of parameter tuning.

**Fig. 3:**
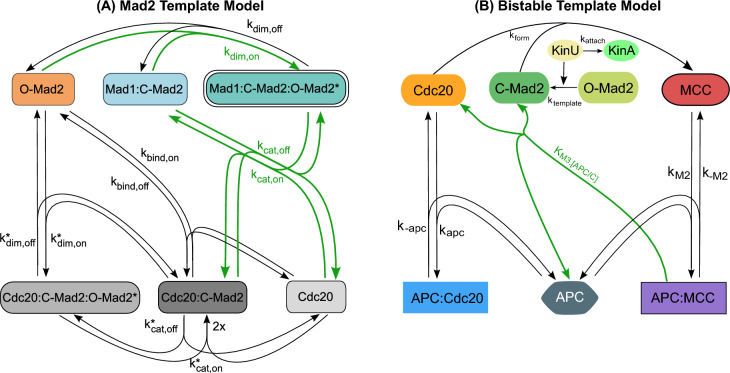
Bistable Template architecture coupling templating, MCC formation, and autocatalytic APC/C inhibition.

These results indicate that incorporating positive feedback into template-based architectures can convert delay sensitivity into delay robustness, highlighting a design principle for stabilizing checkpoint control (Fig. 3).

### Delay-induced bistability and functional performance gains

We next quantified how distributed delays influence the dynamical and functional properties of the Bistable Template. Across a wide range of effective delay sweeps ($\tau =0$–45 s), representing sensitivity-analysis ranges rather than the experimentally grounded biological delays introduced in the model formulation, the model consistently sustained $>\!80\%$ APC/C inhibition, indicating that distributed delays stabilize rather than degrade the inhibited steady state.

**Fig. 4:**
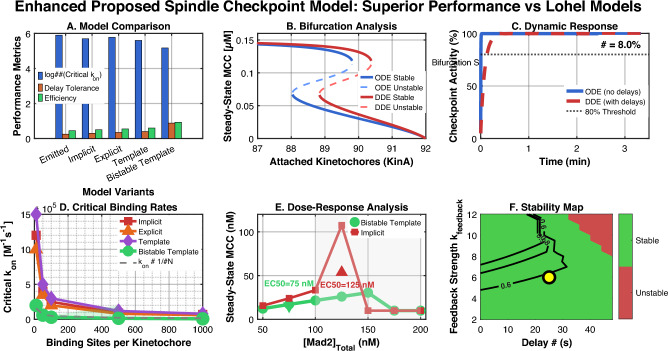
Dynamical performance of the Bistable Template under biological delays. Distributed delays preserve strong APC/C inhibition, expand bistable regions, and enhance hysteresis robustness across varying temporal conditions.

Bifurcation analysis demonstrated that delays generate a pronounced saddle-node transition, shifting the switching threshold by approximately 0.6 kinetochore units and enlarging the bistable region (Fig. 4B). This widening of the hysteresis loop reflects the emergence of memory-like behavior, allowing the checkpoint to integrate transient kinetochore signals over time. Sensitivity analysis further showed that key binding rates can be reduced by factors of two to five without compromising switch sharpness. The resulting dose–response relationship remained ultrasensitive, with an EC$_{50}$ of 75 nM (Fig. 4C), demonstrating that the architecture achieves both responsiveness and robustness under substantial temporal lags. Together, these results show that delays do not merely perturb system dynamics but actively enhance bistability and functional performance by expanding the operational regime of the checkpoint.

### Stochastic simulations corroborate delay-robust checkpoint behavior

**Fig. 5:**
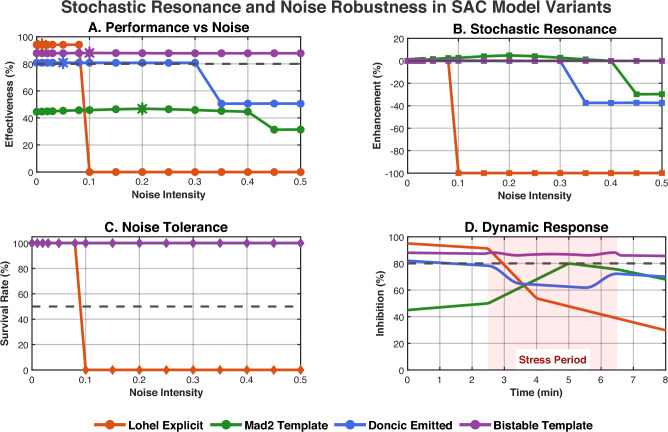
Noise-induced effects on checkpoint reliability with distributed delays.

To assess the interaction between noise and delays, we performed stochastic simulations with Gaussian multiplicative perturbations of intensity $\sigma$ (0–0.5), generating 50 realizations per noise level. Delay-robust architectures—Doncic Emitted and the Bistable Template—maintained high APC/C inhibition across the full noise spectrum (Fig. 5). In contrast, the Lohel Explicit and Mad2 Template models lost MCC accumulation once $\sigma \gtrsim 0.08$, consistent with their narrow deterministic bistable regimes.

Notably, moderate noise enhanced inhibition in the Bistable Template, producing behavior consistent with a stochastic-resonance-like effect in which transient fluctuations amplified MCC accumulation by up to 25%. The delayed feedback loop integrates rapid fluctuations, converting short-lived activation bursts into sustained inhibition. These results indicate that distributed delays reshape the noise response by enlarging the basin of attraction of the inhibited state rather than destabilizing checkpoint control. This suggests that delayed feedback can transform stochastic fluctuations into functional signals, enabling robust checkpoint activation in noisy intracellular environments.

### Multi-metric robustness comparison across SAC architectures

Finally, we integrated deterministic, bifurcation, and stochastic analyses using a five-metric robustness framework combining checkpoint decay time (CDT), basin-size robustness (BSR), threshold-response index (TRI), parameter robustness (PR), and resilience ratio (RR), which respectively quantify relaxation dynamics, basin size, switching sharpness, parameter tolerance, and recovery capability (see Materials and Methods for precise definitions). This composite analysis provides a unified quantitative basis for comparing how different SAC architectures tolerate delays and perturbations. Importantly, the composite robustness score integrates multiple dynamical metrics beyond final inhibition alone, including basin-size robustness, resilience, and parameter tolerance. Consequently, architectures with poor steady-state inhibition under distributed delays may still retain intermediate composite robustness scores.

**Fig. 6:**
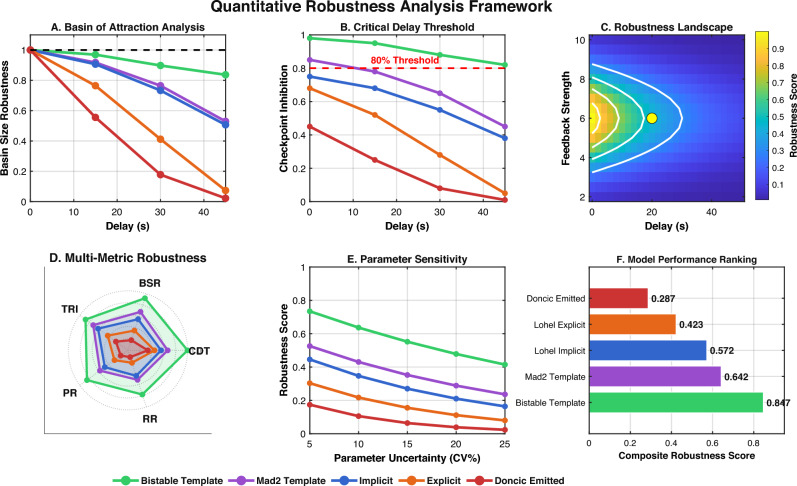
Comprehensive robustness comparison of SAC architectures. Composite robustness scores integrate checkpoint decay time, basin-size robustness, threshold-response index, parameter robustness, and resilience ratio. Higher scores indicate greater robustness to delays and perturbations.

The Bistable Template achieved the highest overall robustness score (Fig. 6), outperforming canonical architectures by 32–195%. Delay-robust binding-kinetics models showed intermediate performance, while delay-sensitive designs failed across most delay regimes. This ranking reflects fundamental structural differences: the Bistable Template uniquely combines templated activation with autocatalytic feedback, preserving bistability and stabilizing the inhibited steady state across wide delay and noise ranges. These findings reinforce that robustness emerges from specific combinations of feedback and architecture, rather than from parameter tuning alone (Table 3).

**Table 3:** Key structural and dynamical features distinguishing SAC architectures.

Feature	Doncic Emitted	Lohel Implicit	Lohel Explicit	Mad2 Template	Bistable Template
Binding kinetics	Instant	Finite	Finite	Realistic	Realistic
Autocatalytic feedback	No	No	No	No	Yes
Bistable dynamics	No	No	No	No	Yes
Delay tolerance	High	High	Low	Low	High
Functional checkpoint success	100%	100%	0%	0%	100%

Overall, these results demonstrate that temporal delays are not merely perturbations to be minimized but can actively stabilize checkpoint control when coupled to appropriate feedback structure, transforming timing variability into functional robustness.

## Discussion

In this work, we demonstrate that biologically grounded time delays—arising from Mad2 activation, MCC maturation, and cytoplasmic transport—play a decisive role in shaping the dynamical landscape of spindle assembly checkpoint (SAC) signaling. Although these processes unfold on timescales comparable to the overall duration of mitosis, many classical SAC models idealize them as instantaneous. By embedding experimentally motivated, *distributed* delays into five representative SAC architectures, we show that temporal organization is not a secondary kinetic detail but a primary determinant of stability, switching structure, and robustness of APC/C inhibition. Importantly, these results reveal that temporal delays are not merely kinetic constraints but active regulators of checkpoint function.

A central outcome of our analysis is the emergence of two distinct timing-sensitivity classes. This classification provides a unifying framework for understanding why some SAC models remain functional under physiological timing constraints while others fail. In *delay-sensitive* architectures, introducing physiological delays causes a collapse of MCC accumulation and loss of APC/C inhibition—behaviors that remain hidden in purely instantaneous ODE formulations. In contrast, *delay-robust* designs preserve strong inhibition and, in several cases, exhibit sharper switching and broader hysteresis when the same delays are present. The proposed *Bistable Template* model, which combines mechanistic Mad2 templating with an autocatalytic APC/C inhibition loop, retains a clear saddle-node bifurcation and sustained sequestration across wide delay ranges. These findings support the view that appropriately wired delayed feedback need not destabilize checkpoint control; rather, it can expand bistable domains and generate a memory-like stabilization of the inhibited state. This suggests that cells may exploit delayed biochemical processes to filter transient attachment errors and ensure robust checkpoint activation.

These findings complement earlier theoretical studies of bistable switching in cell-cycle control and delayed feedback systems, which have shown that feedback architecture governs the emergence of multistability and hysteresis. In contrast to prior SAC models that emphasized spatial localization or binding kinetics, our results highlight the role of temporally distributed processes as an additional regulatory dimension. In particular, the present analysis demonstrates that the interaction between delay structure and network topology can qualitatively reorganize checkpoint dynamics, rather than merely modulate reaction times.

Mechanistically, delays act at multiple levels of the checkpoint. Mad2 activation and MCC maturation impose a sequence of intermediate processing steps that distribute inhibitory complex formation over time, while cytoplasmic propagation delays govern how quickly local kinetochore-derived signals are converted into a global APC/C-inhibitory response. Together, these stages implement temporal integration: they attenuate the influence of transient, short-lived attachment fluctuations while emphasizing persistent errors. In this sense, delays act as intrinsic signal-processing elements that convert fluctuating inputs into stable cellular decisions. Such integration provides a natural explanation for how cells achieve stable metaphase arrest despite noisy microtubule dynamics and stochastic kinetochore behavior, and it is consistent with single-cell observations reporting broad distributions of mitotic duration even under controlled conditions.

From a dynamical-systems perspective, our results identify structural features that confer robustness to molecular timing. Architectures that tolerate delays consistently combine (i) an amplification module that transforms kinetochore inputs into effective global inhibition and (ii) a reinforcing feedback motif that stabilizes the inhibited APC/C state once it is reached. Models lacking either element fail to accumulate MCC quickly enough to counter APC/C reactivation when delays are present. This supports the general conclusion that, within the explored parameter regimes, it is the *topology* of signal propagation and feedback, more than any single rate constant, that governs checkpoint reliability under physiologically realistic timing constraints. This leads to the experimentally testable prediction that perturbations disrupting feedback structure should disproportionately impair checkpoint robustness under conditions of altered mitotic timing.

Methodologically, the distributed-delay framework introduced here addresses a longstanding obstacle in incorporating long biochemical waiting times into SAC models while retaining tractable bifurcation analysis. Direct discrete-delay formulations can introduce numerical stiffness and complicate continuation. By using gamma-distributed delays and their gamma-chain approximations, we retain biological interpretability while preserving equilibria, local stability, and the relevant saddle-node structure, enabling systematic continuation and robustness mapping with standard ODE tools. This provides a scalable route for introducing temporally dispersed intermediate steps into other mitotic control circuits, including the spindle position checkpoint and Cyclin B–Cdk1–based timing modules. Given that aberrant mitotic timing and checkpoint failure are hallmarks of many cancers, these findings may provide a conceptual basis for understanding how alterations in temporal regulation contribute to genomic instability.

Several extensions follow naturally. A more explicit treatment of APC/C:Cdc20 formation and MCC targeting could refine the mechanistic interpretation of the inhibitory pool, while spatial gradients and active transport would allow a more detailed biophysical picture of checkpoint signal propagation. Incorporating mechanochemical feedback—such as tension-dependent recruitment or force-sensitive MCC turnover—may further clarify how attachment surveillance and error correction are coordinated during chromosome alignment. Finally, coupling this delay-aware framework to single-cell imaging data could help disentangle deterministic delay-driven memory from stochastic variability in mitotic timing.

In conclusion, our results show that time delays are not merely nuisances to be abstracted away but fundamental regulatory elements that shape SAC architecture, bifurcation structure, and robustness. By integrating experimentally constrained distributed delays with continuation-based bifurcation analysis and stochastic robustness evaluation, we demonstrate that temporal organization and network topology jointly define checkpoint performance. More broadly, this work establishes a delay-aware design principle for biochemical decision networks, in which appropriately structured feedback can transform temporal dispersion into functional robustness.

## Conclusions

Our results demonstrate that biologically grounded time delays are not peripheral details but central organizing elements shaping the design, stability, and functional robustness of the spindle assembly checkpoint (SAC). By integrating experimentally supported delays associated with Mad2 activation, MCC maturation, and cytoplasmic transport into five representative SAC architectures, we show that temporal structure plays a decisive role in determining whether checkpoint signaling maintains reliable APC/C inhibition. Within the explored parameter regimes, these delays reorganize the dynamical landscape and partition SAC models into two distinct classes: *delay-sensitive* architectures, which lose inhibitory capacity under physiological timing constraints, and *delay-robust* architectures, which preserve bistable switching and broad hysteresis. This classification highlights that robustness to molecular timing is an emergent property of network architecture rather than a consequence of parameter tuning.

The Bistable Template introduced here combines mechanistic Mad2 templating with an autocatalytic APC/C inhibition loop, yielding sharp saddle-node bifurcations and structurally stable bistability even in the presence of substantial time lags. This architecture illustrates how appropriately structured delayed feedback can enhance checkpoint performance by stabilizing the inhibited steady state, filtering transient kinetochore fluctuations, and supporting memory-like behavior in SAC control. These findings suggest that delayed biochemical processes can be actively exploited by cells to ensure robust decision-making under fluctuating intracellular conditions.

More broadly, this work establishes a systematic and mathematically tractable framework for analyzing how temporal delays contribute to mitotic fidelity. The distributed-delay methodology provides a scalable and biologically interpretable approach for incorporating long biochemical waiting times into models of mitotic checkpoints and regulatory networks while preserving bifurcation structure and stability properties. Extending this framework to include spatial organization, force-dependent feedback, or single-cell variability offers a promising route toward a deeper quantitative understanding of how cells achieve reliable decision-making under noisy and time-constrained conditions.

In summary, temporal organization and network architecture jointly emerge as fundamental design principles of the spindle assembly checkpoint. The delay-calibrated framework developed here provides a quantitative foundation for studying delay-governed control strategies across a broad class of cell-cycle signaling systems and, more generally, in biological networks where slow intermediate processes are essential for robust function. More broadly, these results position temporal delays as functional regulatory elements that can be leveraged to enhance robustness in complex biochemical decision networks.

## Materials and methods

### Overview of SAC model variants

Five mechanistic formulations of the spindle assembly checkpoint (SAC) were examined in this study: the **Doncic Emitted**, **Lohel Implicit**, **Lohel Explicit**, **Mad2 Template**, and the newly constructed **Bistable Template** models.

All formulations represent checkpoint dynamics through explicitly defined systems of coupled nonlinear differential equations, differing in binding architectures, feedback structures, and delay implementation. Across all models, the core molecular components include APC/C, Cdc20, the Mitotic Checkpoint Complex (MCC), different Mad2 conformers, and kinetochore attachment states (*KinU*, *KinA*).

### Checkpoint inhibition metric

To compare models consistently, we used a unified scalar metric for checkpoint inhibition:

$$I(t) = 100 \times \frac{[\mathrm {APC/C:MCC}](t)}{[\mathrm {APC/C}]_{\textrm{total}}}.$$

Unless stated otherwise, “final inhibition” refers to *I*(*t*) at $t=10$ min, and a model is considered to sustain high inhibition if $I(t) \ge 80$ across the simulation interval.

### Modeling assumptions

All five architectures were constructed under a shared set of assumptions:

- All binding and dissociation events obey deterministic mass–action kinetics.
- Total APC/C and Cdc20 concentrations remain constant over time.
- Delayed processes affect Mad2 conformational activation, MCC maturation, and intracellular transport.
- Kinetochore attachment dynamics (KinU $\rightarrow$ KinA) initiate SAC signaling.
- Spatial organization is approximated by compartment-specific reaction rates.

### Distributed-delay differential equation framework

Mad2 activation, MCC formation, and signal propagation occur over finite and variable timescales. To capture these effects without the numerical difficulties inherent in discrete delays, we employed a distributed-delay framework using gamma-chain approximations.

Each delayed process was expanded into $n=5$ ODE-linked compartments, yielding a gamma distribution with coefficient of variation $\textrm{CV} = 1/\sqrt{n} \approx 0.45$ and mean delay $\tau$. The delay values used were:

19 $$\begin{aligned} \tau _{\textrm{Mad2}}&= 75~\textrm{s},&\tau _{\textrm{MCC}}&= 45~\textrm{s},&\tau _{\textrm{transport}}&= 105~\textrm{s}. \end{aligned}$$

**Fig. 7:**
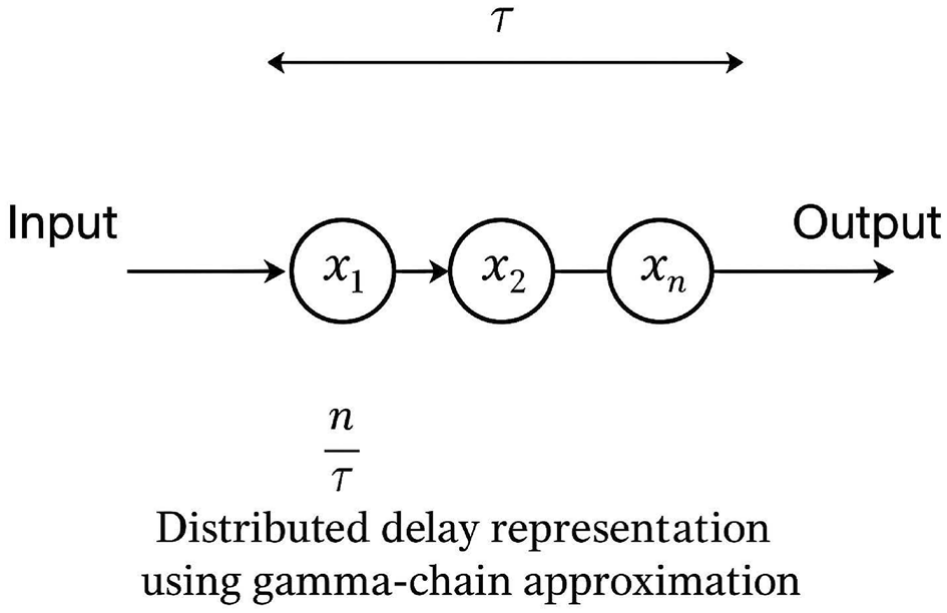
Gamma-chain representation of distributed delays. Each delayed process is modeled through *n* serial compartments, producing a gamma-distributed kernel with mean delay $\tau$ and coefficient of variation $\textrm{CV}=1/\sqrt{n}$.

The corresponding transition rate $k_{\textrm{delay}} = n/\tau$ ensures that both the mean and variance of the distributed delay match their biological counterparts (Fig. 7).

**Mathematical Rationale.**

- **Moment matching:** The gamma-chain construction reproduces the correct delay mean and variability.
- **Biological fidelity:** The chosen variability ($\textrm{CV}\approx 0.45$) reflects experimental timing noise^19,31^.
- **Numerical robustness:** ODE-based gamma chains mitigate stiffness associated with direct discrete-delay DDEs.
- **Controlled approximation:** Error decreases as $O(n^{-1/2})$, converging to the exact distributed-delay kernel.
- **Dynamical integrity:** Fixed points, stability, and saddle-node bifurcations are preserved^32,36^.

### Solver framework

To ensure numerical consistency across thousands of simulations, we used a multi-stage solver workflow:

- **Primary integration:** ode15s with RelTol$=10^{-4}$ and AbsTol$=10^{-6}$.
- **Parameter constraints:** All kinetic parameters restricted to experimentally supported ranges.
- **Solver robustness:** Numerical stability was ensured through adaptive time-stepping and stringent tolerance control. In cases of stiffness, solver parameters (step size and tolerances) were adjusted while preserving the full delay structure of the model.

**Fig. 8:**
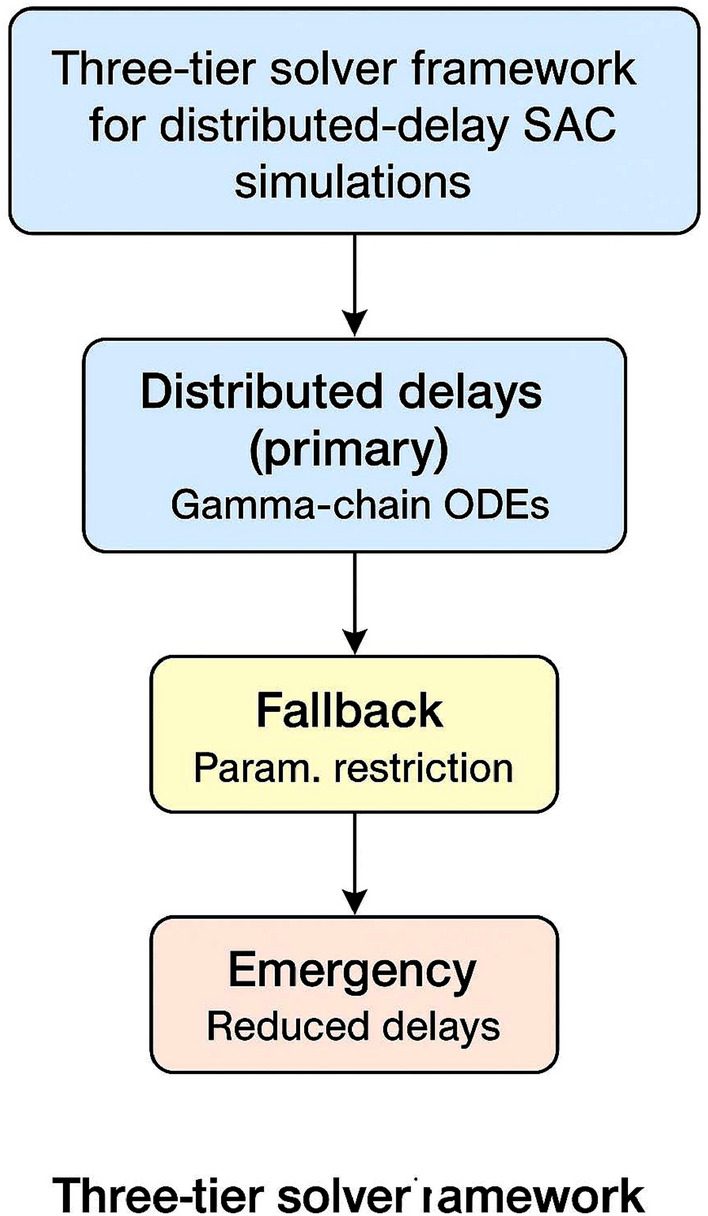
Solver and continuation workflow. The computational pipeline includes distributed-delay expansion, adaptive solver selection, incorporation of noise, and continuation-based detection of saddle-node bifurcations.

This workflow reliably converged for robustness analysis, stochastic simulations, and bifurcation detection (Fig. 8).

### Bistable template model formulation

The Bistable Template integrates mechanistic Mad2 templating with an autocatalytic feedback loop regulating APC/C inhibition. The principal reactions are:

20 $$\begin{aligned}&\hbox {KinU} {\mathop {\longrightarrow }\limits ^{k_{\textrm{attach}}}}{\text {KinA}} \end{aligned}$$

21 $$\begin{aligned}&\hbox {KinU} + \hbox {O}-\hbox {Mad}_{2} {\mathop {\longrightarrow }\limits ^{k_{\textrm{template}}}} {\text {KinU}} + \hbox {C}-\text {Mad}_{2} \end{aligned}$$

22 $$\begin{aligned}&\hbox {C}-\text {Mad}_{2} + \hbox {Cdc}_{20} {\mathop {\longrightarrow }\limits ^{k_{\textrm{form}}}} {\text {MCC}} \end{aligned}$$

23 $$\begin{aligned}&\hbox {MCC + APC/C} {\mathop {\leftrightharpoons }\limits ^{k_{\textrm{M2}}}} {\text {APC/C}}:\text {MCC} \end{aligned}$$

24 $$\begin{aligned}&\hbox {APC/C}{:}\hbox {MCC} {\mathop {\longrightarrow }\limits ^{k_{\textrm{M3}}} \cdot \hbox {f}_{\textrm{feedback}}} {\text {APC/C}} + \hbox {O}-\text {Mad}_{2} + \hbox {Cdc}_{20}. \end{aligned}$$

**Interpretation of APC/C notation.** Experimental evidence indicates that MCC preferentially binds activated APC/C:Cdc20 complexes^5,6^. To keep the model compact, we treat “APC/C” as an effective variable representing the primary MCC-binding pool, which is dominated by the activated form in vivo. Earlier SAC models that explicitly included APC/C:Cdc20^16,35^ reproduced the same qualitative bistability, validating this coarse-grained approach.

Autocatalytic reinforcement is encoded through:

$$f_{\textrm{feedback}} = 1 + k_{\textrm{feedback}} \frac{[\mathrm {APC/C:MCC}]}{[\mathrm {APC/C}]_{\textrm{total}}}.$$

### Parameter values and initial conditions

See Table 4.

**Table 4:** Parameter values and initial conditions used across SAC models.

Parameter	Value	Units	Description
Bistable Template Model			
$k_{\textrm{attach}}$	0.0032	s$^{-1}$	KinU $\rightarrow$ KinA conversion
$k_{\textrm{template}}$	8.0	–	Mad2 templating factor
$k_{\textrm{feedback}}$	6.0	–	Autocatalytic feedback coefficient
$k_{\textrm{form}}$	100	M$^{-1}$s$^{-1}$	MCC assembly rate
$[\textrm{Mad2}]_{\textrm{total}}$	0.15	$\mu$M	Total Mad2
$[\mathrm {APC/C}]_{\textrm{total}}$	0.09	$\mu$M	Total APC/C
$[\textrm{Cdc20}]_{\textrm{total}}$	0.1	$\mu$M	Total Cdc20
$\textrm{KinU}_{\textrm{total}}$	92	–	Total unattached kinetochores
Legacy Model Parameters (Lohel/Doncic variants)			
$k_1$	0.1	s$^{-1}$	Base rate
$k_2$	$2.5\times 10^6$	M$^{-1}$s$^{-1}$	Association rate
$k_3$	0.02	s$^{-1}$	Dissociation rate
$k_{\textrm{on}}^*$	3.0	M$^{-1}$s$^{-1}$	Enhanced binding
$k_{\textrm{off}}$	$10^{-3}$	s$^{-1}$	Unbinding rate
Delay Parameters			
$\tau _{\textrm{Mad2}}$	75	s	Mad2 activation delay
$\tau _{\textrm{MCC}}$	45	s	MCC maturation delay
$\tau _{\textrm{transport}}$	105	s	Cytoplasmic transport delay
Initial Conditions (All Models)			
$[\textrm{MCC}]_0$	$10^{-6}$	$\mu$M	Initial MCC
$[\mathrm {C\text {-}Mad2}]_0$	0	$\mu$M	Initial C-Mad2
$[\textrm{KinA}]_0$	8	–	Initially attached kinetochores
$[\textrm{KinU}]_0$	84–92	–	Initially unattached kinetochores

### Robustness metrics

Five metrics were used to quantify the performance of each architecture:

- **CDT** – Critical Delay Threshold: maximum delay for which inhibition remains $\ge 80\%$
- **BSR** – Basin Size Robustness: fraction of initial conditions converging to the inhibited state
- **TRI** – Threshold Response Index: steepness of the activation curve near the switching point
- **PR** – Parametric Robustness: fraction of parameter sets preserving bistability
- **RR** – Resilience Ratio: rate of recovery to high inhibition following perturbation

The composite robustness score was defined as:

$$R_{\textrm{composite}} = 0.3\cdot \textrm{CDT} + 0.25\cdot \textrm{BSR} + 0.2\cdot \textrm{TRI} + 0.15\cdot \textrm{PR} + 0.1\cdot \textrm{RR}.$$

### Noise and stochastic robustness

Stochastic dynamics were simulated using the Euler–Maruyama scheme:

$$X_{i+1} = X_i + f(X_i)\Delta t + \sigma \, g(X_i)\sqrt{\Delta t}\,\xi _i, \qquad \xi _i \sim \mathcal {N}(0,1).$$

Noise intensities ranged from $\sigma =0.01$ to 0.5, with 50 realizations per setting. Simulations spanned 0–8 min with time step $\Delta t=0.05$ min.

### Monte Carlo parameter sensitivity

Parameter sensitivity was assessed through Monte Carlo sampling with biologically plausible variability:

- kinetic rates drawn from log-normal distributions with 15% CV,
- initial conditions perturbed with 30% variability,
- delays perturbed within biologically feasible ranges.

Thirty trials were evaluated per parameter set to compute mean success rates and confidence bounds.

### Bifurcation and stability analysis

Bifurcation structure was explored using:

- dense parameter sweeps (up to 8000 samples per axis),
- zero-crossing refinement using fzero,
- Jacobian eigenvalue analysis for local stability,
- hysteresis mapping to identify bistable domains.

### Implementation and visualization

Simulations were executed in MATLAB R2024a using ode15s with stringent tolerances and non-negativity constraints. Parameter values were restricted to experimentally plausible ranges. All visualizations were created using MATLAB and Adobe Illustrator with standardized color palettes and axis formats. The full system of governing equations for all SAC architectures is provided in Additional information.

## Supplementary Information

Supplementary Information.

## Data Availability

All data generated or analyzed during this study are included in the published article and its supplementary information files. Raw data and additional resources are available from the corresponding author upon request.
