# Supplementary material for: Network architecture determines delay robustness in the spindle assembly checkpoint
Source: Sci Rep. 2026 Jul 14;16:22078. doi: 10.1038/s41598-026-61538-y (PMC13369184; doi:10.1038/s41598-026-61538-y)
Supplement: Supplementary file 1 — Supplementary Information. [file 41598_2026_61538_MOESM1_ESM.pdf]

## Additional information: Full Model Equations

This additional information provides the explicit systems of ordinary differential equations (ODEs) for all spindle assembly checkpoint (SAC) architectures analyzed in this study. All models follow mass–action kinetics and share common conservation laws.

### A.1 Core Variables and Conservation Laws

Across all models, we track:

- $C$ — free Cdc20
- $M$ — mitotic checkpoint complex (MCC)
- $A$ — free APC/C
- $A_M$ — inhibited APC/C (APC/C:MCC)

with conservation relations:

$$A_{\text{tot}} = A + A_M, \tag{25}$$
$$C_{\text{tot}} = C + M + A_M. \tag{26}$$

### A.2 Doncic Emitted Model

$$\frac{dM}{dt} = k_1 \cdot \text{KinU} \cdot C - k_2 M, \tag{27}$$
$$\frac{dA_M}{dt} = k_3 MA - k_4 A_M, \tag{28}$$
$$\frac{dA}{dt} = -k_3 MA + k_4 A_M, \tag{29}$$
$$\frac{dC}{dt} = -k_1 \text{KinU} C + k_2 M. \tag{30}$$

### A.3 Lohel Implicit Model

$$\frac{dM}{dt} = k_{\text{on}} C - k_{\text{off}} M, \tag{31}$$
$$\frac{dA_M}{dt} = k_{\text{bind}} MA - k_{\text{unbind}} A_M, \tag{32}$$
$$\frac{dA}{dt} = -k_{\text{bind}} MA + k_{\text{unbind}} A_M, \tag{33}$$
$$\frac{dC}{dt} = -k_{\text{on}} C + k_{\text{off}} M. \tag{34}$$

### A.4 Lohel Explicit Model

$$\frac{dK}{dt} = k_1 CA - k_2 K, \tag{35}$$
$$\frac{d(K:E)}{dt} = k_3 K - k_4 (K:E), \tag{36}$$
$$\frac{dM}{dt} = k_5 K - k_6 M, \tag{37}$$
$$\frac{dA}{dt} = -k_1 CA + k_2 K, \tag{38}$$
$$\frac{dC}{dt} = -k_1 CA + k_2 K. \tag{39}$$

### A.5 Mad2 Template Model

$$\frac{dM^*}{dt} = k_{\text{temp}}\text{KinU}(M_{\text{tot}} - M^*) - k_{\text{form}}M^*C, \tag{40}$$

$$\frac{dM}{dt} = k_{\text{form}}M^*C - k_{\text{bind}}MA + k_{\text{unbind}}A_M, \tag{41}$$

$$\frac{dA_M}{dt} = k_{\text{bind}}MA - k_{\text{unbind}}A_M, \tag{42}$$

$$\frac{dA}{dt} = -k_{\text{bind}}MA + k_{\text{unbind}}A_M, \tag{43}$$

$$\frac{dC}{dt} = -k_{\text{form}}M^*C. \tag{44}$$

### A.6 Bistable Template Model

$$\frac{dM^*}{dt} = k_{\text{temp}}\text{KinU}(M_{\text{tot}} - M^*) - k_{\text{form}}M^*C, \tag{45}$$

$$\frac{dM}{dt} = k_{\text{form}}M^*C - k_{\text{bind}}MA + k_{\text{unbind}}A_M, \tag{46}$$

$$\frac{dA_M}{dt} = k_{\text{bind}}MA - k_{\text{unbind}}A_M - k_{\text{fb}}A_M, \tag{47}$$

$$\frac{dA}{dt} = -k_{\text{bind}}MA + k_{\text{unbind}}A_M + k_{\text{fb}}A_M, \tag{48}$$

$$\frac{dC}{dt} = -k_{\text{form}}M^*C + k_{\text{fb}}A_M. \tag{49}$$

### A.7 Incorporation of Distributed Delays

For each model, delayed processes (Mad2 activation, MCC maturation, and transport) are implemented using gamma-chain variables as described in the Mathematical Framework. Specifically, variables such as $M^*$ and $M$ are replaced by their delayed counterparts obtained from $n$-stage linear chains:

$$x_\tau(t) \approx x_n(t),$$

ensuring consistency with the distributed-delay formulation while preserving the finite-dimensional ODE structure.

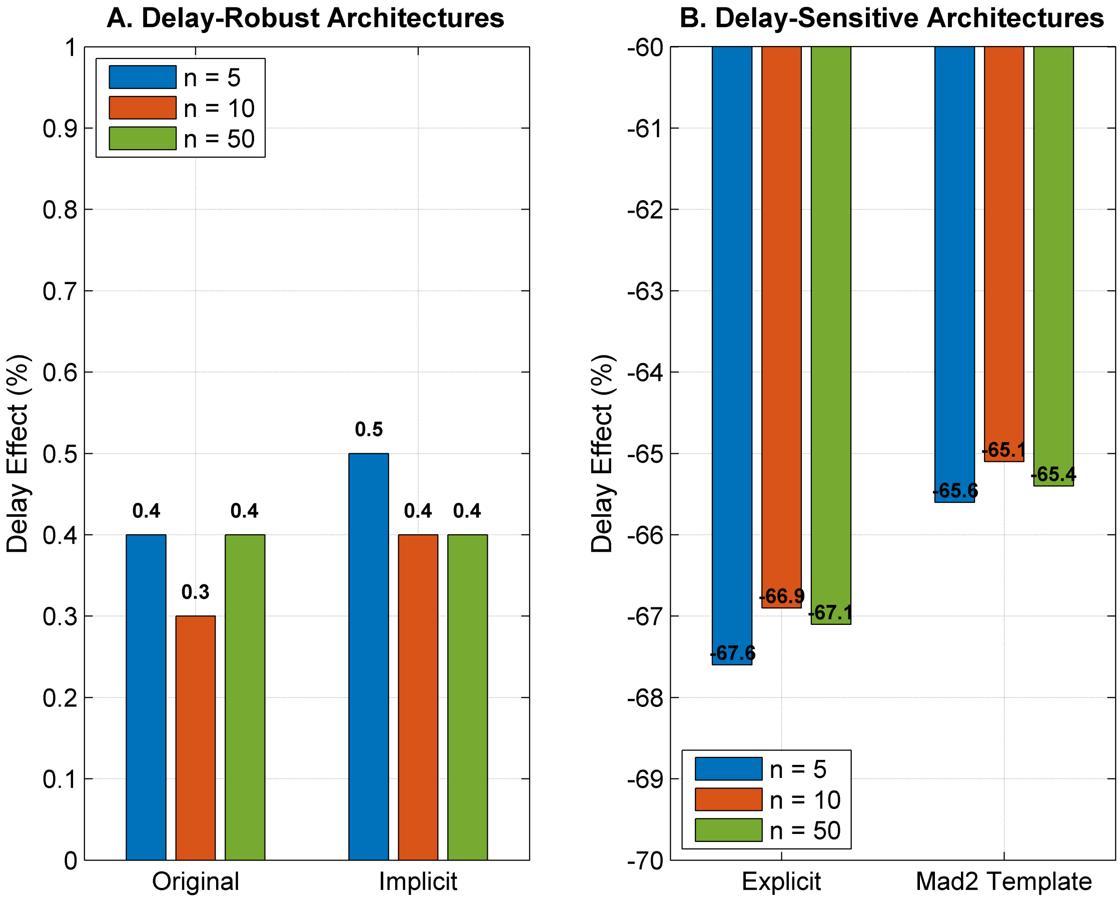


**Figure 9.** **Supplementary Figure S1. Robustness of the architecture classification to the gamma-chain order.** Delay effects obtained for gamma-chain orders $n = 5$, $n = 10$, and $n = 50$. (A) Delay-robust architectures (Doncic Emitted and Lohel Implicit) maintain delay effects close to zero for all tested values of $n$. (B) Delay-sensitive architectures (Lohel Explicit and Mad2 Template) retain large negative delay effects as $n$ increases. These results demonstrate that increasing the gamma-chain order does not alter the qualitative classification of SAC architectures and therefore confirms the robustness of the conclusions with respect to the distributed-delay approximation.

**Table 5.** **Supplementary Table S1. Delay effects for different gamma-chain orders.**

| Model | n=5 | n=10 | n=50 |
|---|---|---|---|
| Doncic Emitted | 0.4% | 0.3% | 0.4% |
| Lohel Implicit | 0.5% | 0.4% | 0.4% |
| Lohel Explicit | -67.6% | -66.9% | -67.1% |
| Mad2 Template | -65.6% | -65.1% | -65.4% |
